# Prevalence of Molecular Markers of Resistance to Antimalarial Drugs Three Years After Perennial Malaria Chemoprevention in Sierra Leone

**DOI:** 10.12688/gatesopenres.16367.1

**Published:** 2025-10-08

**Authors:** Haily Chen, Kwabena Owusu-Kyei, Augustin E. Fombah, Julian Williams, Carla García-Fernández, Eduard Rovira-Vallbona, Andreu Bofill, Llorenç Quintó, Antía Figueroa-Romero, Falama Mac-Abdul, Mohamed Samai, Alfredo Mayor, Clara Menéndez

**Affiliations:** 1 ISGlobal, Barcelona, Spain; 2 Facultat de Medicina i Ciencies de la Salut, Universitat de Barcelona, Barcelona, Spain; 3 Centro de Investigacion Biomedica en Red de Epidemiologia y Salud Publica, Madrid, Community of Madrid, Spain; 4 University of Sierra Leone College of Medicine and Allied Health Sciences, Freetown, Western Area, Sierra Leone; 5 Ministry of Health, Freetown, Sierra Leone; 6 Centro de Investigação em Saude de Manhiça, Manhiça, Maputo Province, Mozambique; 7 Department of Physiological Sciences, Faculty of Medicine, Universidade Eduardo Mondlane, Maputo, Mozambique; 8 Servicio de Salud Internacional, Hospital Clínic de Barcelona, Barcelona, Spain

**Keywords:** malaria, chemoprevention, resistance, IPTi, PMC, U5, sulfadoxine-pyrimethamine, Sierra Leone

## Abstract

**Background:**

Monitoring parasite resistance to antimalarial drugs is essential for detecting potential changes in drug efficacy. This study assessed the prevalence of molecular markers of resistance to sulfadoxine-pyrimethamine (SP), chloroquine, and artemisinin in Sierra Leone, where SP is used for intermittent preventive treatment in pregnancy (IPTp) and perennial malaria chemoprevention (PMC) in young children, while artemisinin is used to treat malaria episodes.

**Methods:**

A cross-sectional survey was conducted between June and August 2021 in three districts of Sierra Leone. A total of 440 febrile children aged 2-5 years attending the health facilities were screened for *P. falciparum* malaria using a rapid diagnostic test, and 300 participants with positive RDT were enrolled. Capillary blood samples were collected as dried blood spots, analyzed using quantitative PCR to confirm *P. falciparum,* and sequenced for resistance markers in *pfdhfr, pfdhps, pfcrt, pfmdr1,* and *pfK13.*

**Results:**

Of 298 blood samples, 237 (79.5%) were qPCR-positive and 230 samples were successfully genotyped. The *pfdhfr* triple mutant (N51I/C59R/S108N) was detected in 99.5% of samples (217/218), while *pfdhps* mutations A437G and K540E were detected in 92.1% (211/229) and 19.1% (42/220), respectively. The *pfdhfr/dhps* quintuple mutant (triple mutant + A437G/K540E) prevalence was 4.6% (7/151), and no sextuple mutants (quintuple + *pfdhps*-A581G) were observed. Chloroquine resistance-associated mutations in *pfcrt* (CVIET haplotype) were detected in 36.6% of samples, while *pfmdr1* mutations at codon 86, 184, 1042, and 1246 occurred in 2.3%, 71.7%, 0.9% and 1.8%, respectively. No validated *pfK13* markers of artemisinin resistance were detected.

**Conclusion:**

In this study, the sustained low prevalence of *pfdhfr/dhps* quintuple mutant justifies the continued use of SP- containing IPTp and PMC, as well as its expansion in the country into the second year of life with additional SP doses. Importantly, no validated *pfK13* markers were found supporting the use of artemisinin-based combination therapies in Sierra Leone.

**Trial registration:**

Clinicaltrials.gov NCT04235816. Registered on January 17, 2020

## Introduction

Malaria accounted for an estimated 246 million cases and 569,000 deaths globally in 2023. ^1^ The African region contributed 95% of malaria cases and 96% of malaria-related deaths, with children under five years of age (U5) being disproportionately affected. ^1^ Effective treatment and prevention of *Plasmodium falciparum* infections with antimalarial drugs are critical for malaria control and elimination. To treat *P. falciparum* malaria, the WHO recommends six artemisinin-based combination therapies (ACTs): artemether-lumefantrine (AL), artesunate-amodiaquine (ASAQ), artesunate-mefloquine (ASMQ), dihydroartemisinin-piperaquine (DP), artesunate-sulfadoxine/pyrimethamine (ASSP), and artesunate-pyronaridine (AP). ^2^

Chemoprevention is a key strategy for malaria control in areas with stable transmission. The WHO recommends three chemoprevention approaches involving sulfadoxine-pyrimethamine (SP) administered at curative doses, irrespective of infection status: intermittent preventive treatment in pregnancy (IPTp), seasonal malaria chemoprevention (SMC), and intermittent preventive treatment in infants (IPTi), now referred to as Perennial Malaria Chemoprevention (PMC). ^2^ SP remains the preferred drug for IPTp, PMC, and SMC (the latter co-packaged with amodiaquine) due to its safety, tolerability, and cost-effectiveness in reducing malaria-related morbidity and neonatal mortality. ^3, 4^ Experiences of SP resistance in Southeast Asia regularly raised concerns about its use for malaria prevention in Africa. ^5^

Pyrimethamine and other antifolates target *P. falciparum* dihydrofolate reductase (DHFR), while sulfadoxine and other sulfonamides act on dihydropteroate synthase (DHPS). Resistance arises from single nucleotide polymorphisms (SNPs) in the *dhfr* and *dhps* genes. Mutations in *dhfr* at codons 51, 59, 108, and 164 confer resistance to pyrimethamine, while SNPs in *dhps* at codons 437, 540, 581, and 613 drive resistance to sulfadoxine. ^6– 8^ These mutations typically accumulate stepwise, leading to stronger drug resistance and the *dhfr/dhps* quintuple mutant (51I, 59R, 108N + 437G, 540E) has been associated with SP treatment failure. ^9, 10^ On the other hand, a pooled study suggested that in areas where the sextuple mutant (quintuple + *dhps* 581G) is present, the effectiveness of IPTp with SP may decline when the prevalence of this mutation exceeds 10%. ^11^

Currently, validated resistance markers for other antimalarial drugs include some single nucleotide polymorphisms (SNPs) in the *kelch 13* gene, providing partial resistance to artemisinin, ^12^ and the K76T in the *pfcrt* gene ( *P. falciparum* chloroquine-CQ- resistance transporter) regarding chloroquine. ^13^

In Sierra Leone, malaria is endemic with perennial transmission and seasonal peaks occurring from May to October. Over 90% of cases are caused by *P. falciparum*, with pregnant women and U5 being the most vulnerable to the infection. ^14^ Malaria prevalence in U5 measured by rapid diagnostic tests (RDTs), declined from 40% in 2016 to 22% in 2021. ^15, 16^ Malaria-attributed mortality has declined significantly since 2000 in Sierra Leone, driven by free diagnostic testing, widespread deployment of insecticide-treated bed nets, and malaria chemoprevention programs. Nationwide IPTp has been implemented in antenatal clinics since 2014 and PMC has been integrated into the Expanded Program on Immunization (EPI) alongside immunization contacts at 10 weeks, 14 weeks, and 9 months, since 2018. Chloroquine, introduced as the first-line treatment for uncomplicated malaria in the 1940s, was replaced in 2004 with artemether-lumefantrine. ^17^

The main objective of this study is to report on the prevalence of molecular markers associated with SP resistance in Sierra Leone seven and three years after the nationwide implementation of IPTp and PMC, respectively. As a secondary objective, we also assessed the prevalence of molecular markers associated with resistance to chloroquine and artemisinin. ^17^

## Methodology

### Study design and sample size

The study was designed as a cross-sectional, health facility-based survey conducted in three districts of Sierra Leone’s Northern Province: Tonkolili, Bombali, and Port Loko. A sample size of 300 children was calculated to detect an ≥8.4% increase in SP resistance prevalence, assuming a baseline prevalence of 10% and accounting for a 10% potential sample loss. ^15^

### Participants recruitment and sample collection

From June to August 2021, children attending outpatient departments of five health facilities in the study area were screened for eligibility. Children who met the following criteria were invited to participate in the study: (i) aged two to five years old, (ii) an axillary temperature ≥37.5 °C or a history of fever in the preceding 24 hours, iii) no signs of severe malaria, ^2^ iv) and a positive result for malaria with an HPR (histidine-rich protein) 2-based rapid diagnostic test (RDT) (Malaria Ag P.f/Pan, SD Bioline ^TM^, Gyeonggi-do, Republic of Korea). The RDT used in this study is listed in the WHO-prequalified in vitro Diagnostic Products used by the Sierra Leone National Malaria Control Program. ^18^

After written informed consent was obtained from caretakers, a questionnaire was administered to collect socio-demographic information and clinical information. Enrolled children underwent clinical assessments, including anthropometric measurements (weight, height/length, and mid-upper arm circumference [MUAC]). Finger-prick blood samples were collected on Whatman ^®^ FTA filter papers in the form of Dried Blood Spots (DBSs), labeled with unique identifiers, were dried completely for 24 hours at room temperature, and stored at 4°C with silica gel until shipment to the Hospital Clínic in Barcelona where they were stored at -20°c until further molecular analysis.

### Laboratory procedures

Parasite genomic DNA was extracted from one 5 mm diameter punch from DBS using a Tween-Chelex-based protocol Merck, Ref: P1379 and C7901, ^19^ eluted in 100 ul of water, and subsequently quantified via qPCR targeting the pf18S ribosomal RNA (rRNA). ^20, 21^ Sequencing of genetic markers of interest ( *pfdhps*, *pfdhfr*, *pfcrt*, *pfmdr1*, and *pfK13*) was performed using the MAD4HatTeR multiplex amplicon sequencing panel (Paragon Genomics Inc, California, USA, Ref: PDG268), ^22^ following previously described procedures. ^23^ Libraries were paired-end sequenced in a NextSeq 2000 system with P1 reagents (Illumina, ref. 20050264). Fastq files were analyzed with MAD4HatTeR Nextflow-based pipeline version 0.1.8 ^24^ using default parameters and the *P. falciparum* 3D7 genome as the reference for alternative allele calling, with the exception of *pfdhps*-A437G where the reference allele G was considered mutant. ^25^ Alleles with fewer reads than the maximum observed in any locus for negative controls were removed, along with alleles with a <1% within-sample frequency. Reconstruction of *pfdhps* double, *pfdhfr* triple and *pfdhfr/pfdhps* quintuple haplotypes was done for samples with no mixed genotypes at selected loci, to minimize phasing complexities.

### Statistical analysis

Descriptive statistics summarized participant characteristics, with continuous variables reported as means ± standard deviations (SDs) and categorical variables as frequencies and percentages.

Crude and multivariate logistic regression models were estimated using Firth’s Penalized Likelihood method to address data separation. ^26, 27^ Profile Penalized-Log Likelihood was used to compute 95% confidence intervals (CIs). Anthropometric z-scores were computed using the LMS method and WHO reference charts, ^28^ with underweight defined as a weight-for-age z-score (WAZ) < -2. Analyses were performed using Stata/SE 18.0, with logistic regression conducted via the *firthlogit* program. ^29^

### Ethics statement

The study protocol and informed consent forms were approved by the Sierra Leone Ethics and Scientific Review Committee (dated August 9, 2020, no approval number) and the Hospital Clínic Research Ethics Committee (Barcelona, Spain) (Registration No: HCB/2020/0173, dated August 28, 2020). The study adheres to the Declaration of Helsinki. The study funder had no role in the design, data collection, analysis, interpretation, or manuscript writing.

### Consent

Written informed consent for publication of the participants details was obtained from the participants’ guardian.

## Results

### Participants characteristics

**Figure 1.**
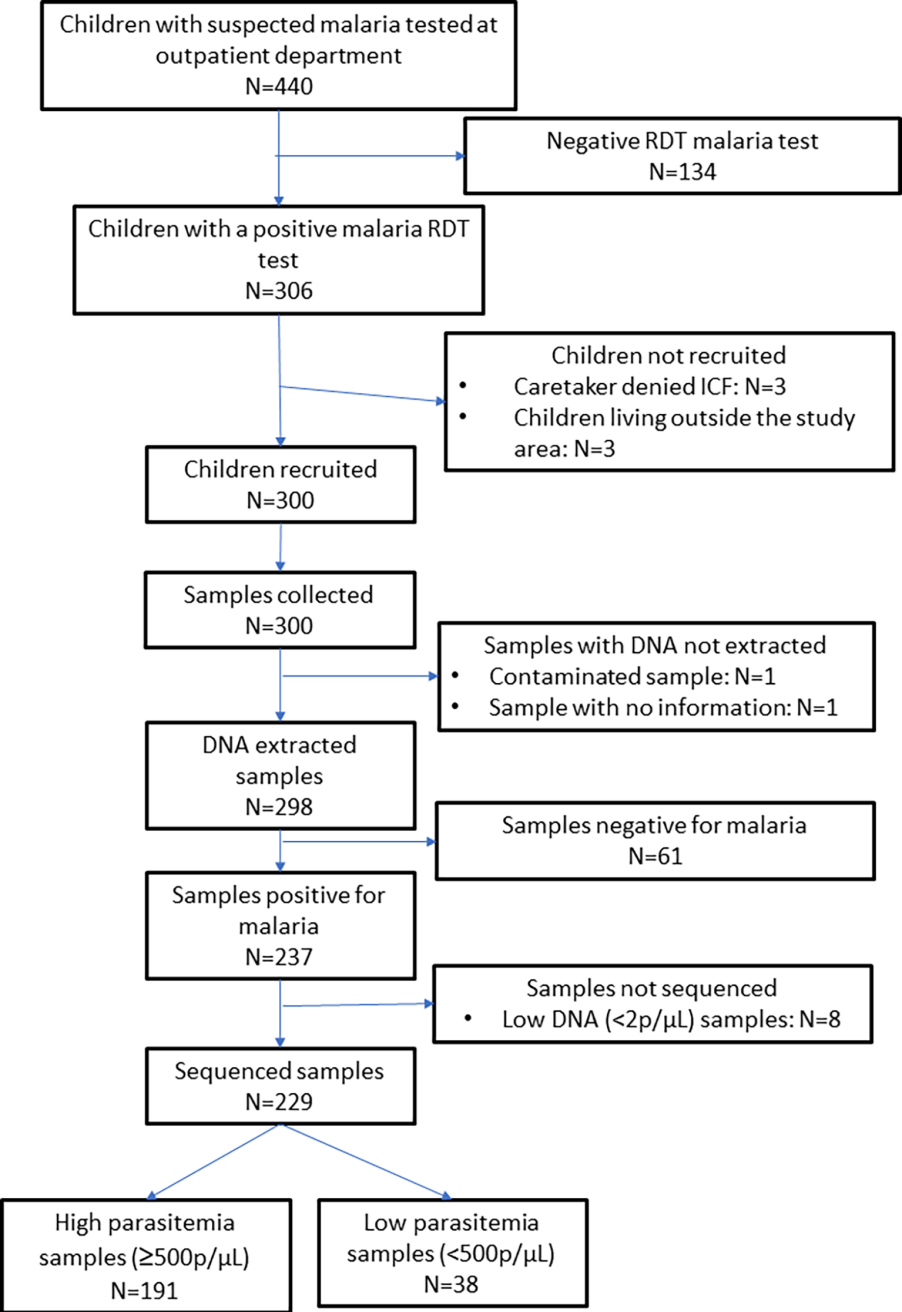
Survey profile. ICF: Informed consent form, RDT: Rapid diagnostic test.

The study was conducted from June to August 2021. Out of 440 children attending the U5 outpatient department with signs and/or symptoms suggestive of malaria, 306 (74.5%) had a positive malaria RDT test. Of them, 300 (98.0%) were enrolled. A capillary sample was collected from these participants for molecular analysis ( Figure 1). The characteristics of the participants are shown in Table 1. The majority of the children were from Tonkolili District (180, 60.0%) and the most common ethnic group was Themne (218, 72.7%). The participants’ mean age was 37.8 months (±10.5) and more than half of the participants (158, 52.7%) were male. A total of 62 (20.7%) children were underweight (defined as having a weight-for-age z-score (WAZ) <-2), and 78 (26.0%) children had an axillary temperature of ≥37.5°C. Most children (211, 70.3%) had slept under a bed net the previous night. A total of 113 (37.7%) children had received an antimalarial within the last month. The mean time since the participants’ last PMC dose was 27.3 months (±10.1).

**Table 1.** Characteristics of study participants.

Variable		n/N	Percentage or mean ± SD [N]
District	Bombali	20/300	6.7%
	Port Loko	100/300	33.3%
	Tonkolili	180/300	60.0%
Age (months)			37.8 (10.5) [300]
Sex	Male	158/300	52.7%
	Female	142/296	47.3%
Ethnic group	Themne	218/296	72.7%
	Mende	22/296	7.3%
	Limba	18/296	6.0%
	Others	42/296	14.0%
Underweight (WAZ <-2)	No	238/300	79.3%
	Yes	62/300	20.7%
Axillary temperature	<37.5°C	222/300	74.0%
	≥37.5°C	78/300	26.0%
High parasitemia (≥500 parasites/μL)	No	38/229	16.6%
	Yes	191/229	83.4%
Bed net use	No	88/300	29.3%
	Yes	211/300	70.3%
	Do not know	1/300	0.3%
Antimalarials received within last month	No	179/300	59.7%
	Yes	113/300	37.7%
	Do not know	8/300	2.7%
On cotrimoxazole (more than 2 weeks)	No	285/300	95.0%
	Yes	8/300	2.7%
	Do not know	7/300	2.3%
Time since last SP dose (months)			27.3 (10.1) [214]

SP: Sulfadoxine-pyrimethamine, WAZ: Weight-for-Age Z-score.

### Molecular markers of resistance to sulfadoxine-pyrimethamine, chloroquine and artemisinin derivatives

Two (0.7%) out of the 300 dried blood spots samples were excluded due to laboratory processing errors. A total of 79.5% (237/298) blood samples were positive for *P. falciparum* by 18S qPCR. Among these, 229 (96.6%) samples were successfully sequenced at *pfdhfr*, and *pfdhps* loci (i.e. allele calls passed both negative controls and allele frequency filters). Of the sequenced blood samples, median parasite density was 13,850 parasites/μL (IQR: 372–79,219), ranging from 0.8 to 737,253 parasites/μL.

Prevalence rates of molecular markers associated to antimalarial drug resistance are presented in Table 2. Six mutations were found in the *pfdhps* gen (I431V, S436A, A437G, K540E and A613S). The single mutant alleles harboring 437A and 540E were found in 211/229 (92.1%; 95% CI 87.9–95.3) of the isolates and in 42/220 (19.1%; 95% CI 14.1–24.9) of isolates, respectively. The double 437/540 mutant allele haplotype was observed in 7/151 (4.6%; 95% CI 1.9–9.3) of isolates.

**Table 2.** Molecular markers of resistance to sulfadoxine-pyrimethamine, chloroquine and artemisinin derivatives.

Gene	Marker	n/N	% (95% CI)
*pfdhps*	I431V	4/229	1.7% (0.5-4.4)
	S436A	125/229	54.6% (47.9-61.2)
	A437G	211/229	92.1% (87.9-95.3)
	K540E	42/220	19.1% (14.1-24.9)
	A581G	0/220	0% (0.0-1.7)
	A613S	47/217	21.7% (16.4-27.7)
	double 437-540 *	7/152	4.6 % (1.9-9.3)
*pfdhfr*	N51I	221/222	99.5% (97.5-100.0)
	C59R	221/222	99.5% (97.5-100.0)
	S108N	222/223	99.6% (97.5, 100.0)
	I164L	0/223	0% (0.0-1.6)
	Triple 51-59-108 *	217/218	99.5% (97.5-100.0)
*Pfdhps/dhfr*	Quintuple mutant *	7/151	4.6% (1.9-9.3)
	Sextuple mutant *	0/196	0%
pfcrt	72-76 CVIET	82/224	36.6% (30.3-43.3)
pfmdr1	N86Y	5/221	2.3% (0.7-5.2)
	Y184F	160/223	71.7% (65.4-77.6)
	S1034C	0/224	0%
	N1042D	2/224	0.9% (0.11-3.2)
	D1246Y	4/226	1.8% (0.48-4,47)
	86-184-1034-1042-1246 NFSND	150/220	68.2% (61.6-74.3)
pfK13	Validated mutations **	0/219	0%

CI: Confidence interval; pfdhfr: Plasmodium falciparum dihydrofolate reductase; pfdhps: Plasmodium falciparum dihydropteroate synthase; pfcrt: Plasmodium falciparum chloroquine resistance transporter; pfmdr1: Plasmodium falciparum multidrug drug resistance gene 1; pfK13: Plasmodium falciparum Kelch 13.

* Only monoallelic infections are included.

** Report on antimalarial drug efficacy, resistance and response: 10 years of surveillance (2010–2019). Geneva: World Health Organization; 2020. License: CC BY-NC-SA 3.0 IGO. ^12^

Regarding mutations in the *pfdhfr* gen *,* three mutations (N51I, C59R, S108N) were detected, with the triple N51I/C59R/S108N mutant allele present in 217/218 (99.5%; 95% CI 97.5-100) of the isolates. The *pfdhps/pfdhfr* quintuple mutant (N51I/C59R/S108N + A437G/K540E) was detected in 7/151 (4.6%; 95% CI: 1.9–9.3) of the isolates. No statistically significant associations were observed between the presence of quintuple mutations to SP and potential host risk factors ( Table 3). No sextuple mutant (N51I/C59R/S108N + A437G/K540E/A581S) was detected in any of the samples analysed.

**Table 3.** Logistic regression of risk factors associated to *P. falciparum* infection with *pfdhps*/ *dhfr* quintuple mutations *.

		Univariable models		Multivariable model	
Variables		Crude odds ratios (95% CI)	P-value	Adjusted odds ratios (95% CI)	P-value
District	Bombali	1	0.7747	1	0.9019
	Port Loko	0.58 (0.02-15.92)		0.59 (0.01-23.05)	
	Tonkolili	1.14 (0.06-23.11)		0.95 (0.04-23.64)	
Age (months)		1.04 (0.97-1.11)	0.3092	1.02 (0.93-1.13)	0.6203
Female		2.62 (0.53-12.94)	0.2360	2.16 (0.45-10.43)	0.3393
Underweight (WAZ <-2)		1.02 (0.16-6.61)	0.9825	0.77 (0.12-5.00)	0.7885
Axillary temperature ≥37.5°C		0.74 (0.12-4.73)	0.7495	0.76 (0.13-4.58)	0.7616
High parasitemia (≥500 parasites/ul)		1.22 (0.19-7.85)	0.8325	1.23 (0.19-7.85)	0.8256
Bed net use		0.45 (0.10-2.08)	0.3052	0.50 (0.11-2.33)	0.3750
Antimalarials received last month		1.34 (0.29-6.23)	0.7048	1.53 (0.29-8.06)	0.6153
Time since last SP dose received (months)		1.05 (0.97-1.13)	0.2254	1.01 (0.93-1.11)	0.7917

SP: sulfadoxine-pyrimethamine, WAZ: Weight-for-Age Z-score.

Penalized maximum likelihood logistic regression.

* n = 101 observations.

The *pfcrt* 72–76 CVIET haplotype that has been associated with *P. falciparum* resistance to chloroquine was detected in 82/224 (36.6%; 95% CI: 30.3-43.3) of the isolates.

Regarding the *pfmdr1* gene, four mutations were detected (N86Y, Y184F, N1042D and D1246Y) in 2.3% (5/221; 95% CI: 0.7-5.2), 71.7% (95% CI: 65.4-77.6), 0.9% (95% CI: 0.11-3.2) and 1.8% (95% CI: 0.48-4.47) of the tested isolates, respectively. No S1034C mutant was detected. Out of the 220 samples with a determinable full haplotype, 61 (27.7%, 95% CI: 21.9-34.1) were wild type for all five loci (NYSND haplotype), and 150 (68.2%; 95% CI: 61.6-74.3) were single mutants (codon 184, NFSND haplotype).

No validated mutations in the *pfk13* gene were detected in any of the 219 sequenced samples ( Table 2).

## Discussion

The study was conducted in Sierra Leone seven and three years after the nationwide implementation of IPTp and PMC, respectively. Since 2015, artemisinin-lumefantrine (AL) is the first-line treatment for uncomplicated malaria in the country. The latest national Malaria Indicator Survey and a household survey from another study, both conducted in 2021, reported that more than half of pregnant women received at least three doses of IPTp, and nearly 60% of infants had received three PMC doses. ^16, 30^ Despite the wide implementation of these SP-based strategies the findings from this study show a low prevalence (4.6%) of the *pfdhfr/pfdhps* quintuple mutant and no presence of the sextuple mutant.

The prevalence of *pfdhfr* triple mutations was nearly fixed (99.5%), consistent with country reports from 2016 and 2018. ^31, 32^ Nevertheless, the prevalence of the *pfdhps/pfdhfr* quintuple mutant was found at a lower prevalence (4.6%) in the current study as compared to the prevalence observed (10%) in the previous survey in 2016, with no data on quintuple mutant reported in 2018. ^32^ The low prevalence of the quintuple mutant in Sierra Leone aligns with rates reported in West Africa, like 15.4% in Guinea (2013-2016), ^33^ 0% in Senegal (2010) ^34^ and 1.6% in Mali (2012). ^35^

The low prevalence of the quintuple mutant and the absence of the sextuple mutant in this study support the continued use of SP for chemoprevention in young children and pregnant women in Sierra Leone. Although the presence of SP resistance markers alone does not determine drug efficacy, especially when used for prevention, sustained drug pressure may drive these mutations toward saturation. ^36^ Therefore, regular monitoring of molecular resistance markers is important to understand how their prevalence affects drug effectiveness and to tailor malaria prevention programs.

In this study, we also assessed the prevalence of molecular markers associated with chloroquine and artemisinin resistance in the country. Mutations in the *pfcrt* gene at codon 72-76 have been associated with *P. falciparum* tolerance to chloroquine and other 4-aminoquinolines such as amodiaquine and piperaquine. The *pfcrt* prevalence reported in this study is higher than the 22% observed in 2018 in a Southern district in the country, though the latter was based on a sample size of only 95 participants. ^37^ The *pfcrt* prevalence varies across West Africa, ranging from the highest in Liberia (87.9%, 2018), ^38^ a moderate level in the Democratic Republic of Congo (22.7%, 2019), ^39^ to low in Equatorial Guinea (2.8%, 2019) ^40^ and Togo (0.6%, 2021). ^41^ Mutations in the *pfmdr1* gene also modulate parasite susceptibility to several ACT partner drugs (e.g. lumefantrine, mefloquine, and piperaquine). The predominant *pfmdr1* NFSND haplotype detected in Sierra Leone is consistent with trends observed across West African countries that adopted artemether-lumefantrine (AL) and discontinued chloroquine as first-line treatment. ^41– 45^ *pfmdr1* mutations exhibit pleiotropic effects, with N86Y driving resistance to chloroquine and amodiaquine while sensitizing parasites to lumefantrine, mefloquine, and artemisinin derivatives. ^46– 49^ These findings underscore the importance of molecular surveillance to optimize ACT use in different regions.

Several mutations in the *pfK13* gene are associated with artemisinin resistance. ^12^ However, none were detected in this study, indicating no molecular evidence of compromised efficacy of artemisinin and its derivatives in Sierra Leone. Nonetheless, the emergence of such mutations has been recently reported in Rwanda, ^50^ Uganda, ^51^ and the Democratic Republic of Congo, ^52^ mirroring the early stages of resistance observed in Southeast Asia. In that region, *P. falciparum* developed partial resistance to artemisinin-based combination therapies (ACT), leading to treatment failures and the spread of resistance strains. ^5^ To prevent a similar trend in Africa, regular surveillance and monitoring of artemisinin resistance are crucial.

Nonetheless, the presence of resistance markers alone does not explain the reduced efficacy of antimalarial drugs. A meta-analysis of seven clinical trials concluded that IPTp with SP reduces placental malaria, low birthweight, and anemia in pregnant women, even in areas with SP treatment failure in children. ^11^ Factors such as patient adherence, metabolism, host immunity, and nutritional status also influence drug efficacy. These factors vary by individual, population, and region, requiring careful interpretation of molecular resistance markers and their impact on chemoprevention. ^5^

This study has some strengths and limitations. A key strength is its geographical coverage, spanning three districts in Sierra Leone’s Northern Province, making it more representative than previous surveys in 2016 and 2018, which were limited to the Kambia or Bo districts. ^31, 32, 37^

The study was limited by the fact that 20.5% (61/298) of blood samples from RDT-confirmed participants tested negative for *P. falciparum* by qPCR. These potential false positives may be due to the residual presence of HRP2 antigens in the bloodstream, which can persist in the blood for up to 28 days after infection clearance. ^53^ In addition, of the 61 participants with negative qPCR results, 28 reported having taken an antimalarial drug within the past month, which would be in line with the false-positive RDT results and better agrees with previously observed false positive rates of 10% with this RDT test. ^54^ Another explanation is a potential DNA degradation during sample preservation.

In 2022, the WHO recommended additional PMC-SP doses to extend protection beyond the first year of life. ^55^ The current findings showing a low prevalence of the *pfdhfr/pfdhps* quintuple mutant and the absence of the sextuple mutant support the continued use of SP for IPTp and PMC, as well as the expansion of PMC with additional SP doses administered in the second year of life in Sierra Leone.

## Data Availability

As the dataset contains potentially identifying information on participants, it is stored under restricted access. For more detailed information beyond the metadata and documentation provided, there is a process of managed access requiring the submission of a request for consideration. Please contact this email for data access: andreu.bofill@isglobal.org Reporting guidelines: The manuscript adheres to STROBE checklist for observational cross-sectional studies.
